# Efficacy of four exercise modalities on body composition in overweight and obese university students: a network meta-analysis of randomized controlled trials

**DOI:** 10.7717/peerj.21418

**Published:** 2026-07-06

**Authors:** Pengfei Lv, Shijie Feng, Shuangtao Xing

**Affiliations:** 1 School of Physical Education, Sias University, Zhengzhou, China; 2 School of General Education, Zhengzhou University of Economics and Business, Zhengzhou, China; 3 School of Physical Education, Henan Normal University, Xinxiang, China

**Keywords:** Exercise modalities, Overweight and obese, University students, Network meta-analysis, Randomized controlled

## Abstract

**Background:**

The rising prevalence of overweight and obesity among university students constitutes a pressing public health concern; however, the differential effectiveness of various exercise interventions on body composition remains insufficiently characterized.

**Methods:**

This network meta-analysis aimed to evaluate the effects of four exercise modalities—high-intensity interval training (HIIT), resistance exercise (RE), aerobic exercise (AE), and combined aerobic-resistance exercise (AE+RE)—on adiposity-related outcomes in overweight and obese university students. The primary outcomes assessed included body mass index (BMI), body fat percentage (BFP), body weight (BW), hip circumference (HC), waist circumference (WC), and body fat mass (BFM). We systematically searched the Data Knowledge Service Platform to identify randomized controlled trials (RCTs) published up to July 1, 2025. Data on study characteristics, intervention details, and outcome measures were extracted, and statistical analyses were performed using RevMan 5.3 and Stata 16 software. A total of 39 RCTs involving 2,585 participants were included in the final analysis.

**Results:**

Compared with the control group, AE+RE emerged as the most effective intervention for improving BMI, BW, and BFM (mean difference (MD) = 2.57 , 95% confidence interval (CI) [1.82–3.32], *P* < 0.001). Aerobic exercise was found to be most effective for reducing WC (MD = 8.44 , 95% CI [1.16–15.72], *P* < 0.001), while RE demonstrated superior efficacy in decreasing HC (MD = 4.78, 95% CI [1.97–7.59], *P* = 0.0009). HIIT yielded the greatest improvements in BFP (MD = 4.05, 95% CI [1.05–7.05], *P* = 0.008).

**Conclusions:**

These findings address the existing gap in knowledge regarding exercise efficacy for overweight and obese university students and provide a scientific foundation for the development of personalized public health strategies.

## Introduction

Overweight and obesity have emerged as major global health challenges in recent decades. Driven by rising living standards and evolving lifestyle patterns, the global prevalence of overweight and obesity has increased steadily (Jastreboff et al., 2018). According to the World Health Organization (WHO), nearly 1 billion people worldwide are currently affected by overweight or obesity, accounting for approximately one-eighth of the global population. Projections suggest that by 2050, this number will reach 3.80 billion—surpassing half of the global adult population at that time (Lobstein et al., 2025).

Recent epidemiological data highlight a particularly alarming trend: the prevalence of obesity among university students has risen sharply and remains higher than that of their age-matched peers, such as adolescents or young adults entering the workforce. University represents a critical period for the formation of long-term lifestyle behaviors, rendering this population a key focus for research on obesity-related factors and the development of targeted public health interventions. Unhealthy lifestyle habits, including physical inactivity and excessive caloric intake, have been identified as primary contributors to overweight and obesity in this group (Newsome et al., 2024).

Exercise intervention stands as a cornerstone strategy for fat reduction, offering the dual benefit of reducing adiposity while improving overall health and physical fitness. Compared with pharmaceutical interventions or dietary restriction, exercise is more accessible, cost-effective, and sustainable. While aerobic exercise (AE) has long been the mainstay of weight management protocols, resistance exercise (RE), high-intensity interval training (HIIT), and combined aerobic-resistance exercise (AE+RE) have gained increasing attention in recent years due to their purported efficacy.

Aerobic exercise refers to physical activity performed under conditions of adequate oxygen supply, typically involving sustained, rhythmic movements. It enhances fat metabolism and induces favorable adaptations in adipose cell size and metabolic (Batrakoulis, 2023). By maintaining exercise intensity at 50–75% of maximum heart rate and extending exercise duration, fat is effectively utilized as an energy source, increasing overall energy expenditure and achieving superior fat reduction outcomes.

Research into the fat-loss effects of HIIT has expanded rapidly in recent years. Characterized by brief bouts of maximal or near-maximal effort followed by short, low-intensity recovery intervals (repeated cyclically) (Leung et al., 2024), HIIT demonstrates fat-loss efficacy comparable to that of traditional long-duration AE. However, its manageable duration and flexible intensity make it more appealing to the general public (Zhang, 2016).

Resistance exercise, also known as strength training, improves body composition by increasing muscle mass, boosting resting metabolic rate, and reducing body fat (Alkahtani et al., 2014). From a physiological perspective, RE enhances post-exercise calorie consumption—a phenomenon known as excess post-exercise oxygen consumption (EPOC)—as the body continues to burn energy during the recovery period. This sustained metabolic boost contributes to effective long-term fat loss (Higgins & Higgins, 2016). The American College of Sports Medicine (ACSM) highlighted “evidence-based resistance training” at its 61st Annual Meeting, noting its ability to provide intense muscle stimulation and high fitness efficiency, positioning it as a future trend in health promotion.

Combined AE+RE has also demonstrated promising effects on body composition. For instance, a 10-week intervention study found that among overweight and obese university students, combined training resulted in greater improvements in body fat percentage (PBF), BMI, and BW compared with AE alone (Zhao & Liang, 2019).

While research into single factors influencing fat loss—such as exercise type, intensity, or duration—has advanced both theoretically and practically (Wu, Wang & Song, 2017), comparative studies investigating how to optimally combine these factors to target specific obesity-related indicators in overweight and obese university students remain limited. To address this gap, we conducted a network meta-analysis to compare the fat-loss effects of four exercise modalities in this population and identify the optimal exercise intervention for each key adiposity indicator.

## Survey methodology

This meta-analysis was reported in accordance with the Preferred Reporting Items for Systematic Reviews and Meta-Analyses (PRISMA) statement. The study protocol was registered in the International Prospective Register of Systematic Reviews (PROSPERO-CRD42024592806) prior to the initiation of formal literature screening against eligibility criteria. Pengfei Lv and Shijie Feng jointly designed and executed the literature search strategy in compliance with PRISMA guidelines. Disagreements during the literature retrieval process were first resolved through discussion between the two investigators; unresolved disputes were referred to Shuangtao Xing, an independent referee, who provided objective judgments based on pre-defined eligibility criteria to ensure methodological rigor.

### Search methodology

**Figure 1:**
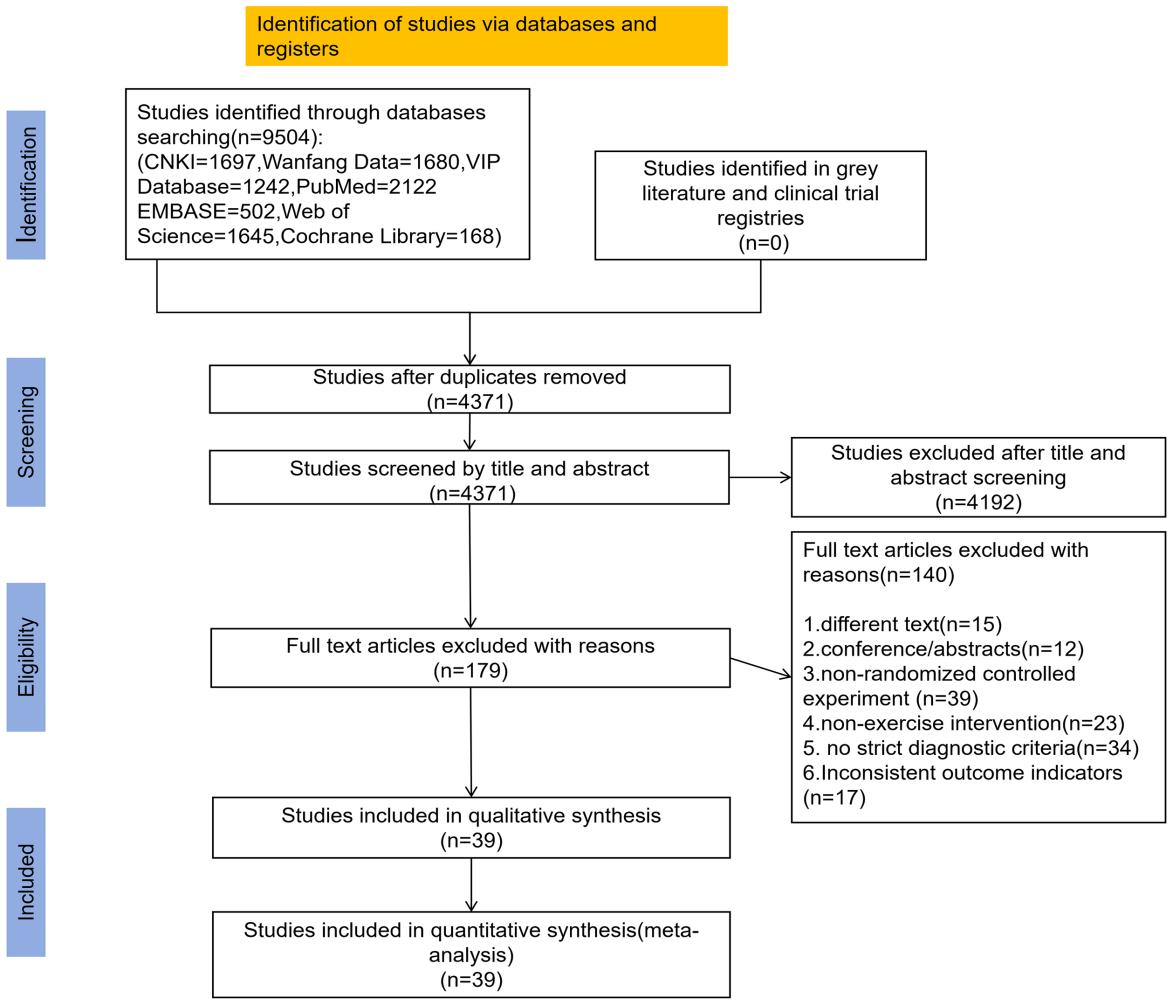
Effects of four exercise modes on obesity. Study selection flow chart according to the preferred reporting items for systematic reviews and meta-analyses (PRisMA) statements.

The Data Knowledge Service Platform (encompassing CNKI, Wanfang, VIP, PubMed, EMBASE, Web of Science, and the Cochrane Library) was searched to identify relevant studies investigating the effects of various exercise interventions on fat loss in overweight and obese university students. A combination of MeSH terms, keywords, and free-text terms was used, with the search timeframe spanning from database inception to July 1, 2025. Key search terms included: “physical activity”, “aerobic exercise”, “HIIT”, “resistance exercise”, “combined aerobic-resistance exercise”, “obesity”, “overweight”, “adiposity”, “excess weight”, “weight loss”, “fat loss”, “young adults”, “undergraduates”, and “university students” (Fig. 1).

### Criteria for inclusion and exclusion

#### Type of research: randomized controlled trials

##### Study population.

Included studies focused on overweight and obese university students (defined as BMI > 24 kg/m^2^) with no major comorbidities, who received one of the following exercise interventions: AE, HIIT, RE, or AE+RE.

##### Interventions.

Experimental groups were assigned to one of the four exercise modalities (AE, HIIT, RE, AE+RE). Control groups (CT) either received no exercise intervention or maintained their routine physical activity levels.

##### Exclusion criteria.

Studies were excluded if they met any of the following criteria: (1) duplicate publications; (2) non-randomized or inadequately randomized study designs; (3) abstract-only publications with no full-text access; (4) studies with unextractable or unanalyzable outcome data.

### Literature screening and data extraction

Two independent investigators conducted a double-blind review to identify eligible studies and extract relevant data, including: author information, publication year, participant demographics (gender, age), intervention details (type, intensity, duration), and outcome indicators (body mass index (BMI), hip circumference (HC), waist circumference (WC), body weight (BW), body fat percentage (PBF), body fat mass (BFM)). Following data extraction, the two investigators cross-checked their results. Discrepancies were resolved through consultation with a third independent researcher, and the final set of included studies was confirmed.

### Quality assessment of included studies

The methodological quality of included studies was evaluated by two independent reviewers using the revised Cochrane Risk of Bias Tool (ROB 2.0). Five key domains were assessed: randomization process, deviations from intended interventions, missing outcome data, measurement of outcomes, and selective reporting of outcomes. A total of nine studies were rated as having “some concerns” in at least one domain, indicating insufficient information to confirm a low risk of bias.

### Statistical analysis

Network meta-analysis was performed using Stata 16 software. First, inconsistency tests were conducted to evaluate closed loops within the evidence network; a *p*-value > 0.05 indicated coherence between direct and indirect comparisons (CDI). Next, probability rankings of interventions were visualized using Surface Under the Cumulative Ranking Curve (SUCRA) plots for outcomes reported in at least three studies. SUCRA values were calculated for all six outcome measures (BMI, BW, WC, HC, PBF, BFM). Cluster analysis was used to determine the optimal exercise intervention for each outcome. The study protocol was registered in the International Prospective Register of Systematic Reviews (PROSPERO-CRD42024592806).

## Results

### Literature screening process and outcomes

Database searches initially identified 9,504 potentially relevant studies. After removing 4,371 duplicates and excluding 1,356 irrelevant studies based on title and abstract screening, 178 studies underwent full-text review. Of these, 39 met the eligibility criteria and were included in the final meta-analysis (Fig. 1, Table 1).

**Table 1:** Basic characteristics of included literature.

Study	Sample size (T/C)	Average age		Intervention measure		Outcome measure
		T	C	T	C	
Ge et al. (2014)	8/8	25.63 ± 1.85	25.13 ± 1.25	AE	Daily activities	①②⑥
He (2012b)	30/30	20.24 ± 1.52	20.24 ± 1.52	AE	Daily activities	①②③④⑥
Lv & Xiong (2019)	10/10	18.31 ± 1.46	18.21 ± 1.33	AE	Daily activities	①②
He (2012a)	30/30	20.24 ± 1.52	20.24 ± 1.52	AE	Daily activities	③④
Zou, Tang & Mo (2005)	10/10	20.3	20.3	AE	Daily activities	⑥
Krishnamoorthi, Kodeeswaran & Senthil (2021)	15/15	18-22	18-22	AE	Daily activities	①
Cui, Lv & Ding (2018)	26/26	18-22	18-22	AE	Daily activities	①②
Guo (2018)	50/50	20.5 ± 1.5	20.5 ± 1.5	AE	Daily activities	①
Xiao et al. (2022)	22/22/22	20.8 ± 1.1	20.9 ± 1.7	AE	Daily activities	①②⑥
		20.8 ± 1.8		AE+RE		
Yu (2018)	30/30	18-22	18-22	AE	Daily activities	①②③④
Yan (2009)	12/12	20.16 ± 1.19	20.02 ± 1.31	AE	Daily activities	①②⑥
Hu et al. (2022)	20/20	19.20 ± 1.10	20.20 ± 0.40	HIIT	Daily activities	①②⑤⑥
Tong et al. (2018)	16/14	18-23	18-23	HIIT	Daily activities	②⑤⑥
Lin et al. (2016)	18/18	20.2 ± 2.1	20.2 ± 2.1	HIIT	Daily activities	①②③④⑤⑥
Roberts, Croymans & Aziz (2013)	28/8	20.8–22.8	20.0–23.0	RE	Daily activities	①③
Qian, Lin & Liu (2020)	8/8	19.63 ± 1.06	19.87 ± 0.82	RE	Daily activities	②③④⑥
Tanimoto et al. (2008)	12/12	19.0 ± 0.6	19.8 ± 0.7	RE	Daily activities	⑥
Chen et al. (0000)	22/23	19.27 ± 0.83	18.7 ± 0.82	RE	Daily activities	①⑤⑥
Ma (2014)	10/10	20.5 ± 2.4	21.4 ± 2.8	RE	Daily activities	①②
Kim et al. (2018)	10/10	22.90 ± 2.23	24.50 ± 1.72	AE+RE	Daily activities	②③⑥
Kang et al. (2012)	6/6	21–23	21–23	AE+RE	Daily activities	②③⑥
Xiao, Zhen & Wang (2020)	30/30	18–23	18–23	AE+RE	Daily activities	①②
Li & Fan (2017)	12/12/12	18–23	18–23	AE	Daily activities	①②⑤⑥
				AE+RE		
Ge (2020)	24/24	21.48 ± 2.85	21.35 ± 3.07	AE+RE	Daily activities	①②③④⑤⑥
Asad et al. (2012)	12/9/13/10	22.0 ± 0.89	21.44 ± 1.13	AE	Daily activities	①②
		21.0 ± 1.57		RE		
		21.38 ± 2.06		AE+RE		
Sun et al. (2020)	150/150	21.78 ± 1.47	21.63 ± 1.39	HIIT	AE	①④
Yang et al. (2022)	12/12	20 ± 2	20 ± 2	HIIT	AE	①②③⑤⑥
Chen, Wu & Su (2021)	12/12	20.57 ± 1.08	20.57 ± 1.08	HIIT	AE	①②③⑤⑥
Kong et al. (2016)	13/13	21.5 ± 4.0	20.5 ± 1.9	HIIT	AE	①②⑤⑥
Tsai et al. (2016)	20/20/20	22.2 ± 1.6	22.4 ± 1.1	AE	Daily activities	①②
		22.3 ± 1.0		HIIT		
Trapp et al. (2008)	15/15/15	22.4 ± 0.7	22.2 ± 0.12	AE	Daily activities	②⑤⑥
		22.4 ± 0.7		HIIT		
Mazurek et al. (2016)	24/24	20.9 ± 0.94	20.9 ± 0.94	HIIT	AE	①②
Wang et al. (2023)	22/21	19.50 ± 1.44	19.33 ± 1.35	HIIT	AE	①②
Lo et al. (2011)	10/10	20.0 ± 0.67	21.1 ± 1.66	RE	AE	①②③④⑤⑥
Chen & Zhang (2022)	10/7	18-25	18-25	HIIT	Daily activities	③
Hovsepian et al. (2021)	30/30	20.23 ± 1.30	20.69 ± 1.70	HIIT	Daily activities	③
Wang, Hang & Zhang (2015)	12/12	21.0 ± 1.0	20.6 ± 1.2	HIIT	AE	①③④
Chen, Yang & Yuan (2019)	30/30	20.98 ± 0.98	21.25 ± 1.24	AE	Daily activities	②⑥
Xu et al. (2025)	21/22	18.48 ± 0.68	18.27 ± 0.77	AE	Daily activities	③

**Notes.**

T, treatment group; C, control group; ①, BMI; ②, WT; ③, WC; ④, HC; ⑤, BFM; ⑥, PBF.

### Characteristics of included studies and risk of bias assessment

**Figure 2:**
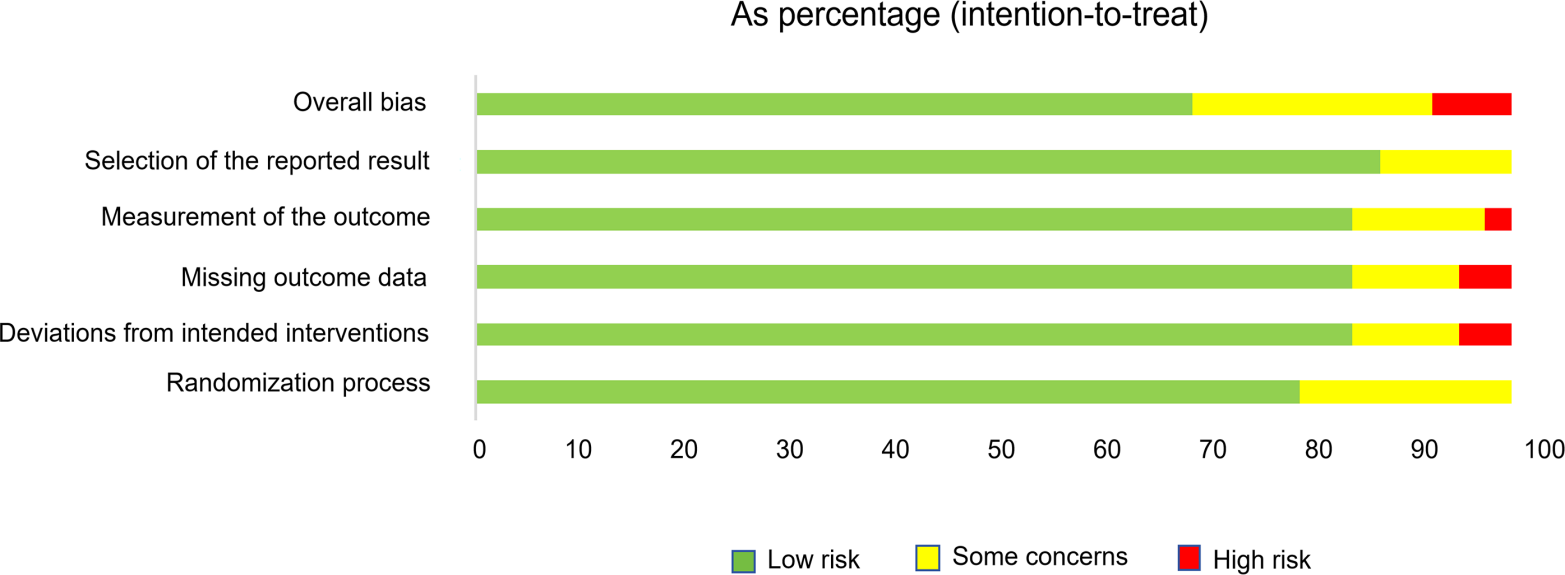
Risk summary of bias in the included literature.

**Figure 3:**
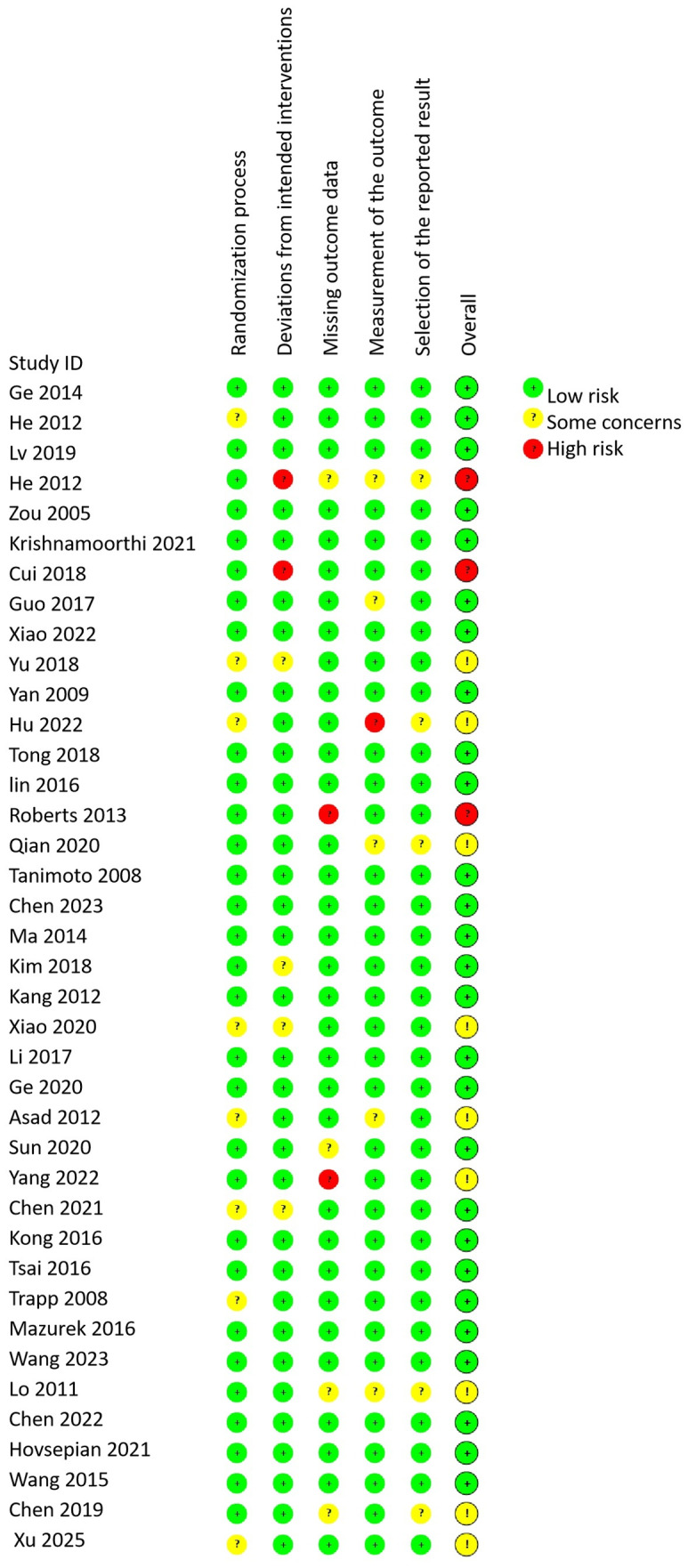
Risk-of-bias assessment according to the revised Cochrane risk-of-bias tool for randomized trials.

Table 1 summarizes the baseline characteristics of the included studies, while Figs. 2 and 3 present the results of the risk of bias assessment. Nine studies were rated as having a “high risk” of bias in at least one domain. Among these, three studies exhibited issues with randomization, suggesting potential flaws in group allocation (Figs. 2 and 3).

### Network meta-analysis results

#### Inconsistency test

Node-splitting analysis revealed that *p*-values for BMI (*P* = 0.2256), BW (*P* = 0.2707), WC (*P* = 0.2991), HC (*P* = 0.8410), BFM (*P* = 0.3674), and PBF (*P* = 0.4388) all exceeded 0.05. These findings confirm coherence between direct and indirect comparisons for all six outcome measures, supporting the reliability of the network meta-analysis results.

#### Body mass index

**Figure 4:**
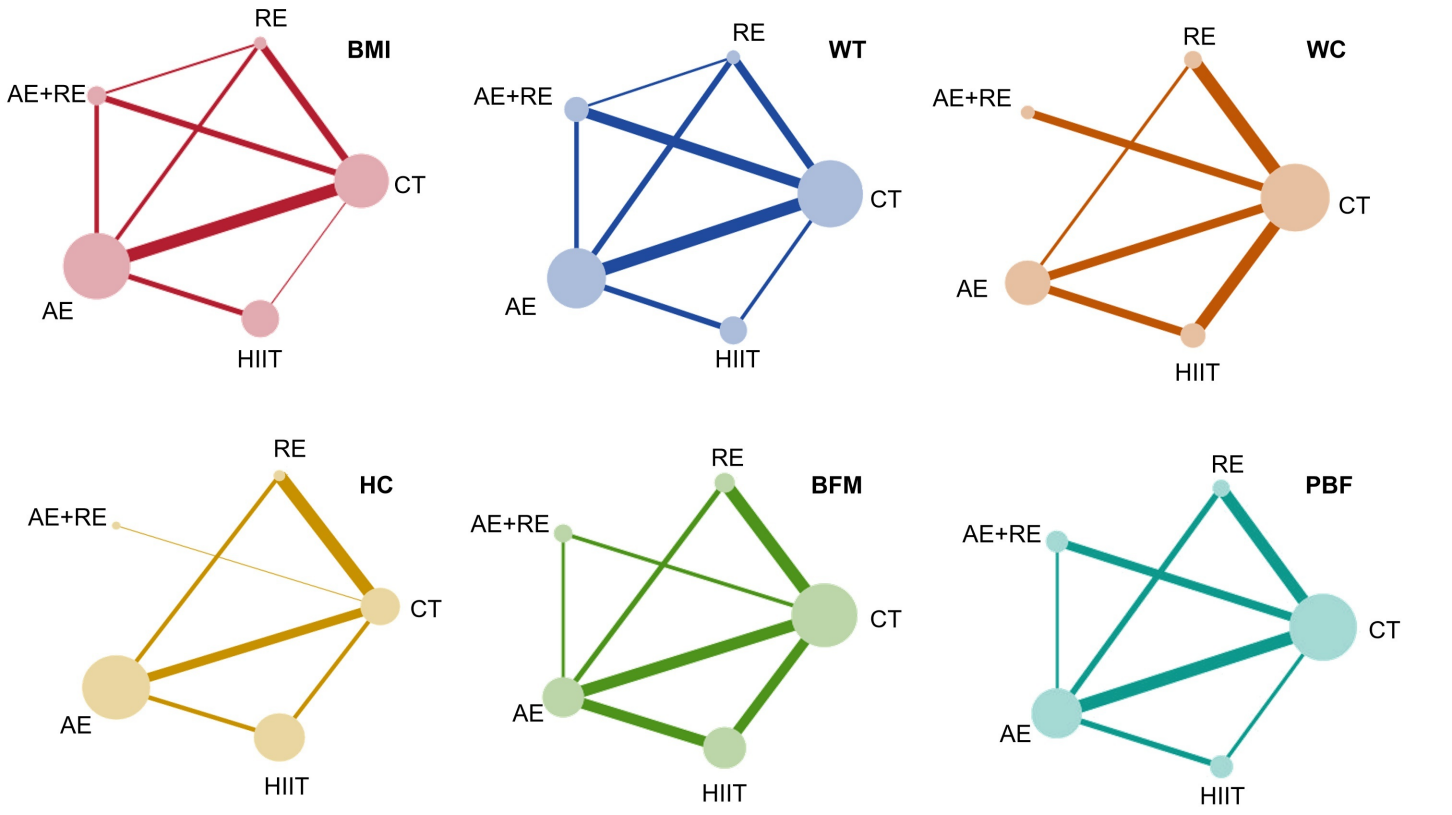
Summary of network geometry of BMI, WT, WC, HC, BFM, PBF.

**Figure 5:**
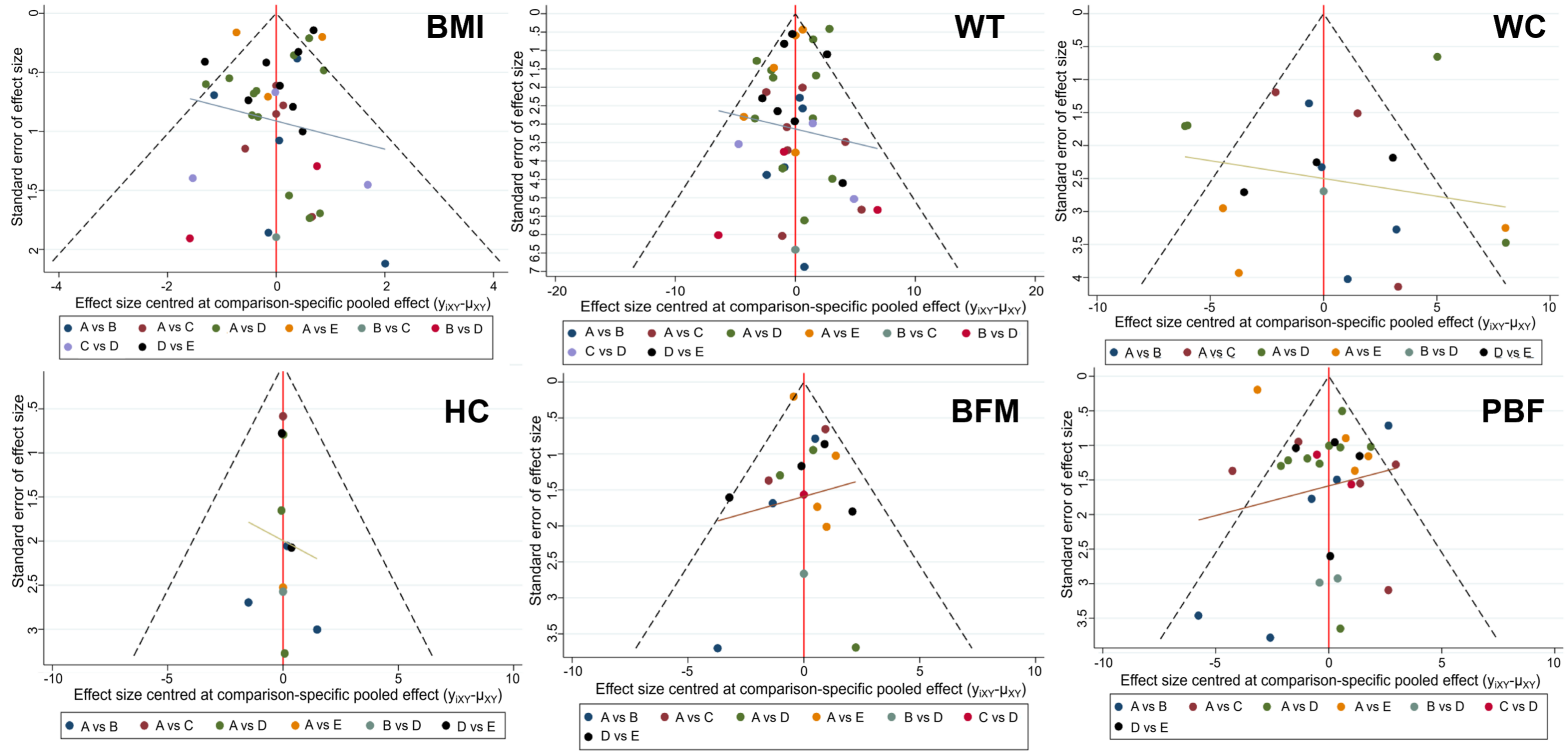
Comparison-correction funnel diagram.

Twenty-eight studies involving 1,403 participants were included in the analysis of BMI outcomes. Network meta-analysis demonstrated that AE (MD = 0.98, 95% CI [0.69–1.26], *P* < 0.001), HIIT (MD = 1.45, 95% CI [0.19–2.70], *P* < 0.001), RE (MD = 0.79, 95% CI [0.18–1.40], *P* < 0.001), and AE+RE (MD = 2.57, 95% CI [1.82–3.32], *P* < 0.001) all significantly improved BMI compared with the control group. Direct comparisons between exercise modalities revealed that AE+RE produced more pronounced improvements than RE (MD = 1.26, 95% CI [0.13–2.39], *P* < 0.001), while HIIT was more effective than AE (MD = 0.52, 95% CI [0.04–0.99], *P* < 0.001) (Figs. 4 and 5). SUCRA probability rankings confirmed AE+RE as the most effective intervention for improving BMI, with RE being the least effective (Tables 2 and 3).

**Table 2:** Probability ranking results of influence of different exercise methods on fat loss of overweight and obese college students.

Intervention measure/ Outcome measure	BMI	WT	WC	HC	BFM	PBF
AE	46.4	47.1	74.0	40.7	62.0	50.8
HIIT	80.2	61.3	66.4	77.1	65.0	73.8
RE	30.2	51.7	58.1	80.9	25.8	65.2
AE+RE	92.7	89.6	49.3	51.3	96.5	60.2
CT	0.7	0.3	2.3	0.0	0.7	0.0

**Notes.**

The larger the value, the better the improvement effect.

**Table 3:** Probability ranking of the effects of different exercise methods on improving various outcome indicators in overweight and obese college students.

**A**	**CT**	3.27 (0.50, 6.05)	4.94 (2.73, 7.15)	3.26 (2.07, 4.44)	3.62 (2.22, 5.03)
	0.83 (−0.03, 1.69)	**RE**	1.67 (−1.80, 5.13)	−0.02 (−2.90, 2.87)	0.35 (−2.68, 3.38)
	2.09 (1.31, 2.87)	1.26 (0.13, 2.39)	**AE+RE**	−1.68 (−4.06, 0.69)	−1.32 (−3.87, 1.24)
	1.26 (0.82, 1.70)	0.43 (−0.51, 1.36)	−0.83 (−1.65, −0.00)	**AE**	0.37 (−0.96, 1.69)
	1.78 (1.24, 2.33)	0.95 (−0.05, 1.94)	−0.31 (−1.22, 0.60)	0.52 (0.04, 0.99)	**HIIT**
**B**	**CT**	4.45 (1.97, 6.92)	3.31 (2.17, 4.45)	3.08 (1.79, 4.37)	4.09 (2.27, 5.90)
	5.22 (0.51, 9.92)	**RE**	−1.14 (−3.86, 1.59)	−1.37 (−4.01, 1.28)	−0.36 (−3.31, 2.59)
	4.35 (−1.40, 10.11)	−0.86 (−8.29, 6.57)	**AE+RE**	−0.23 (−1.95, 1.50)	0.78 (−1.37, 2.92)
	6.40 (2.42, 10.38)	1.19 (−4.43, 6.80)	2.05 (−4.94, 9.03)	**AE**	1.00 (−0.37, 2.38)
	5.93 (1.12, 10.75)	0.72 (−5.75, 7.18)	1.58 (−5.92, 9.08)	−0.47 (−5.12, 4.18)	**HIIT**
**C**	**CT**	3.70 (1.65, 5.75)	3.54 (1.87, 5.20)	3.36 (2.24, 4.47)	3.92 (2.44, 5.41)
	1.90 (−0.05, 3.84)	**RE**	−0.16 (−2.80, 2.47)	−0.34 (−2.60, 1.91)	0.22 (−2.31, 2.75)
	5.71 (4.06, 7.35)	3.81 (1.35, 6.27)	**AE+RE**	−0.18 (−1.98, 1.62)	0.39 (−1.76, 2.53)
	4.12 (2.80, 5.44)	2.23 (−0.16, 4.61)	−1.58 (−3.57, 0.41)	**AE**	0.57 (−0.95, 2.09)
	4.21 (3.01, 5.41)	2.31 (−0.06, 4.69)	−1.50 (−3.51, 0.52)	0.08 (−1.15, 1.32)	**HIIT**

**Notes.**

Note A: the top right part is the result of WT comparison, and the bottom left part is the result of BMI comparison.

Note B: the upper right part is the comparison between HC and WC, and the lower left part is the comparison between WC and HC.

Note C: the upper right part is the comparison between PBF and BFM, the lower left part is the comparison between BFM and PBF.

#### Body weight

Twenty-seven studies involving 1,010 participants were included in the BW analysis. Network meta-analysis showed that AE (MD = 3.58, 95% CI [1.91–5.25], *P* < 0.001), HIIT (MD = 2.50, 95% CI [1.97–3.02], *P* < 0.001), and AE+RE (MD = 7.78, 95% CI [3.57–7.98], *P* < 0.001) all significantly reduced BW compared with the control group. No significant differences were observed in direct comparisons between exercise modalities (*p* > 0.05; Figs. 4–5). SUCRA rankings identified AE+RE as the most effective intervention for BW reduction, with AE being the least effective (Table 2, Fig. 3).

#### Waist circumference

Fifteen studies involving 517 participants were included in the waist circumference (WC) analysis. Network meta-analysis indicated that AE (MD = 8.44, 95% CI [1.16–15.72], *P* < 0.001), RE (MD = 5.21, 95% CI [3.12–7.30], *P* < 0.001), and AE+RE (MD = 4.84, 95% CI [1.73–7.95], *P* = 0.002) all significantly reduced WC compared with the control group. Direct comparisons between exercise modalities revealed no statistically significant differences (*p* > 0.05; Figs. 4 and 5). SUCRA probability rankings confirmed AE as the most effective intervention for WC reduction, with AE+RE being the least effective (Tables 2 and 3).

#### Hip circumference (HC)

Nine studies involving 624 participants were included in the HC analysis. Network meta-analysis demonstrated that AE (MD = 3.07, 95% CI [1.70–4.44], *P* < 0.001), RE (MD = 4.78, 95% CI [1.97–7.59], *P* = 0.0009), and AE+RE (MD = 3.31, 95% CI [2.17–4.45], *P* < 0.001) all significantly reduced HC compared with the control group. No significant differences were observed in direct comparisons between exercise modalities (*p* > 0.05; Figs. 4–5). SUCRA rankings identified RE as the most effective intervention for HC reduction, with AE being the least effective (Table 2, Fig. 3).

#### Body fat mass (BFM)

Eleven studies involving 374 participants were included in the BFM analysis. Network meta-analysis showed that AE (MD = 4.50, 95% CI [3.03–5.97], *P* < 0.001), HIIT (MD = 4.93, 95% CI [4.54–5.31], *P* < 0.001), and AE+RE (MD = 5.81, 95% CI [3.48–8.14], *P* < 0.001) all significantly reduced BFM compared with the control group. Direct comparison revealed that AE+RE was more effective than RE (MD = 3.81, 95% CI [1.35–6.27], *P* < 0.001) (Figs. 4 and 5). SUCRA rankings confirmed AE+RE as the most effective intervention for BFM reduction, with RE being the least effective (Tables 2 and 3).

#### Body fat percentage (PBF)

Twenty studies involving 648 participants were included in the PBF analysis. Network meta-analysis indicated that AE (MD = 3.28, 95% CI [2.64–3.91], *P* < 0.001), HIIT (MD = 4.05, 95% CI [1.05–7.05], *P* = 0.008), RE (MD = 3.31, 95% CI [0.78–5.84], *P* = 0.01), and AE+RE (MD = 3.07, 95% CI [0.36–5.78], *P* = 0.03) all significantly reduced PBF compared with the control group. Direct comparisons between exercise modalities revealed no statistically significant differences (*p* > 0.05; Figs. 4 and 5). SUCRA rankings identified HIIT as the most effective intervention for PBF reduction, with AE being the least effective (Tables 2 and 3).

### Publication bias and small-study effect assessment

Funnel plot analysis for BMI revealed that most data points were symmetrically distributed around the vertical central line. However, six outlier points suggested potential publication bias, and one point at the base of the funnel indicated a potential small-study effect (Fig. 5).

## Discussion

Previous research has consistently identified unhealthy lifestyle habits—including physical inactivity and excessive caloric intake—as primary drivers of overweight and obesity (Dong et al., 2024). Exercise intervention serves as a key strategy for fat reduction, offering benefits beyond weight loss, such as improved physical fitness and overall health. Compared with pharmaceutical interventions or dietary restriction, exercise interventions are more accessible, cost-effective, and sustainable. To our knowledge, this is the first network meta-analysis to systematically compare the effects of four common exercise modalities on body composition in overweight and obese university students. Through probability ranking, we found that AE+RE ranked first for improving BMI, BW, and BFM; AE was most effective for WC reduction; HIIT yielded the greatest improvements in PBF; and RE was superior for HC reduction.

Aerobic exercise emerged as the most effective intervention for WC reduction. Continuous AE enhances metabolic efficiency in obese individuals by promoting fat oxidation for energy production, thereby reducing adipose cell size and achieving fat loss. He (2014) demonstrated that AE not only effectively reduces excess body fat but also improves muscle tone, increases lean muscle mass, and reduces circumferential measurements of various body segments—collectively contributing to healthier body composition. Consistent with numerous prior studies, our findings confirm that regular engagement in moderate-to-low intensity AE (*e.g.*, brisk walking, jogging) combined with targeted abdominal exercises can significantly reduce WC and abdominal fat. Notably, AE exhibited greater efficacy in reducing abdominal fat compared with HIIT, RE, or AE+RE.

Resistance exercise was identified as the most effective intervention for HC reduction. Regular RE has been shown to significantly decrease HC in overweight and obese populations; for example, a study of overweight and obese women found that 12 weeks of RE resulted in a significant reduction in hip circumference (Damas et al., 2015). Resistance exercise improves body composition in overweight and obese individuals by increasing muscle mass, elevating resting metabolic rate, and reducing fat mass—physiological effects that help reduce fat accumulation in the hip region. Additionally, RE may influence HC through indirect mechanisms, such as improved insulin sensitivity, regulated fat metabolism, and reduced adipose cell size—all of which contribute to hip fat reduction.

High-intensity interval training ranked as the most effective intervention for PBF reduction. Compared with traditional AE, HIIT offers greater benefits for reducing visceral fat, primarily due to increased total energy expenditure—particularly during the post-exercise recovery phase (EPOC). This post-exercise energy expenditure is proportional to exercise intensity and exhibits gender differences: men tend to experience higher EPOC, while women may require longer exercise durations to achieve comparable effects. Our meta-analysis confirms that HIIT is effective in reducing PBF among overweight and obese university students, consistent with the findings of Dai et al. (2023) and Cao et al. (2022). While moderate-to-low intensity AE burns significant amounts of fat during exercise, HIIT results in greater total energy expenditure due to sustained post-exercise fat oxidation. Furthermore, HIIT exerts a more pronounced appetite-suppressing effect than moderate-intensity AE, which may help control energy intake and further support fat loss.

Combined AE+RE emerged as the top-ranked intervention for improving BMI, BW, and BFM. Aerobic exercise enhances cardiovascular function and energy metabolism, while RE strengthens muscles, bones, and joints—improving flexibility, balance, and range of motion, and reducing injury risk. A large body of evidence indicates that AE+RE significantly improves health status and exercise capacity, with this combined approach demonstrating superior BW loss outcomes in overweight and obese populations (Smart et al., 2024). The synergistic effects of AE and RE likely contribute to its enhanced efficacy: AE promotes acute fat oxidation, while RE increases lean muscle mass and resting metabolic rate—supporting long-term fat loss and weight maintenance.

### Study limitations

This study has several limitations that should be considered when interpreting the results. First, the included participants exhibited variability in age and duration of obesity; thus, the efficacy of different exercise interventions for overweight and obese university students across different age groups and obesity durations requires further investigation. Second, the included studies varied in terms of participant race, country, region, and gender, and no subgroup analyses were performed to explore these moderating factors. Future research should include high-quality, large-sample, multi-center RCTs to investigate the effects of age, obesity duration, race, and geographic region on exercise intervention efficacy in this population—ultimately improving the personalization and effectiveness of exercise recommendations.

## Conclusion

In conclusion, scientifically designed exercise regimens are effective for fat loss in overweight and obese university students. Based on our network meta-analysis, we recommend AE+RE for improving BMI, BW, and BFM; AE for WC reduction; HIIT for PBF reduction; and RE for HC reduction. Overweight and obese university students should enhance their health literacy, develop awareness of evidence-based fat loss strategies, and select exercise types and durations that align with their individual characteristics to improve exercise adherence and long-term fat loss outcomes.

## Supplemental Information

10.7717/peerj.21418/supp-1 Supplemental Information 1 Raw data.

10.7717/peerj.21418/supp-2 Supplemental Information 2 PRISMA checklist.

## Additional Information and Declarations

### Data Availability

The following information was supplied regarding data availability: This is a systematic review/meta-analysis.
